# Phylogenetic Grouping of Human Ocular *Escherichia coli* Based on Whole-Genome Sequence Analysis

**DOI:** 10.3390/microorganisms8030422

**Published:** 2020-03-17

**Authors:** Konduri Ranjith, Chinthala Reddy SaiAbhilash, Gumpili Sai Prashanthi, Shalem Raj Padakandla, Savitri Sharma, Sisinthy Shivaji

**Affiliations:** Jhaveri Microbiology Centre, Prof. Brien Holden Eye Research Centre, L. V. Prasad Eye Institute, Kallam Anji Reddy campus, Hyderabad, Telangana 500034, India; konduriranjit@gmail.com (K.R.); abhilashreddy@lvpei.org (C.R.S.); saiprashanthi.g@lvpei.org (G.S.P.); shalemraj@lvpei.org (S.R.P.); savitri@lvpei.org (S.S.)

**Keywords:** AMR, *E. coli*, ocular, whole genome analysis, phylogenetics, pathotype

## Abstract

*Escherichia coli* is a predominant bacterium in the intestinal tracts of animals. Phylogenetically, strains have been classified into seven phylogroups, A, B1, B2, C, D, E, and F. Pathogenic strains have been categorized into several pathotypes such as Enteropathogenic (EPEC), Enterotoxigenic (ETEC), Enteroinvasive (EIEC), Enteroaggregative (EAEC), Diffusely adherent (DAEC), Uropathogenic (UPEC), Shiga-toxin producing (STEC) or Enterohemorrhagic (EHEC) and Extra-intestinal pathogenic E. coli (ExPEC). *E. coli* also survives as a commensal on the ocular surface. However, under conditions of trauma and immune-compromised states, *E. coli* causes conjunctivitis, keratitis, endopthalmitis, dacyrocystitis, etc. The phylogenetic affiliation and the pathotype status of these ocular *E. coli* strains is not known. For this purpose, the whole-genome sequencing of the 10 ocular *E. coli* strains was accomplished. Based on whole-genome SNP variation, the ocular *E. coli* strains were assigned to phylogenetic groups A (two isolates), B2 (seven isolates), and C (one isolate). Furthermore, results indicated that ocular *E. coli* originated either from feces (enteropathogenic and enterotoxigenic), urine (uropathogenic), or from extra-intestinal sources (extra-intestinal pathogenic). A high concordance was observed between the presence of AMR (Antimicrobial Resistance) genes and antibiotic resistance in the ocular *E. coli* strains. Furthermore, several virulent genes (*fimB* to *fimI*, *papB* to *papX*, etc.) and prophages (Enterobacteria phage HK97, Enterobacteria phage P1, *Escherichia* phage *D108* etc.) were unique to ocular *E. coli.* This is the first report on a whole-genome analysis of ocular *E. coli* strains.

## 1. Introduction

The human eye is sterile prior to birth, but immediately after birth the ocular surface acquires several bacteria from the mother and the environment, and these survive on the ocular surface as commensals. The ocular surface commensal bacteria include *Pseudomonas aeruginosa*, *Staphylococcus aureus*, *Staphylococcus epidermidis*, *Micrococcus luteus*, *Neisseria* spp., *Moraxella* spp., *Bacillus* spp., *Rhodococcus erythropolis, Propionibacterium acnes*, *Klebsiella oxytoca*, *Escherichia coli*, *Proteus mirabilis*, *Enterobacter agglomerans*, *Klebsiella* spp. etc. More recently, based on 16S rRNA gene sequencing, a greater bacterial diversity has been observed associated with the lid margin and lower conjunctival sac [1,2,3]. These bacteria are normally harmless and do not cause any infections. However, under conditions of trauma and when the host’s immunity is compromised, these commensal bacteria become infective and cause diseases like conjunctivitis, keratitis, or endopthalmitis [4,5,6]. In general, transformation from a commensal to a virulent form depends on several attributes, such as the ability to swim, adhere, form a biofilm, produce toxins, and avoid host defense mechanisms [7]. Virulence is also enhanced by virulent factors [8], the presence of prophages, plasmids, ability to conjugate, etc. [9]. These studies also suggested that transposons, plasmids and insertion sequences contribute to the plasticity of the *E. coli* genome, resulting in an extremely large pangenome [10]. 

The origin of ocular *E. coli* is not very clear, but ocular microbiologists believe that they most likely originate from fecal or urine contamination or from an extra-intestinal source. It is possible that these ocular *E. coli* are related to one or more of the known eight pathotypes of *E. coli*, namely enteropathogenic (EPEC), enterotoxigenic (ETEC), enteroinvasive (EIEC), enteroaggregative (EAEC), diffusely adherent (DAEC), uropathogenic (UPEC), Shiga-toxin producing (STEC), or enterohemorrhagic (EHEC) and extra-intestinal pathogenic *E. coli* (ExPEC) [11]. Phylogenetically, these ocular *E. coli* could be affiliated with the already characterized phylogenetic groups designated A, B1, B2, C, D, E, and F [12,13,14,15,16,17], and strains affiliated to B2 and D often carry virulence determinants that are lacking in group A and B1 strains [15,17]. In a recent study, Raimondi et al. [12] demonstrated that the ExPEC strains residing as commensals in the guts of healthy subjects mostly belonged to phylogroup B2, followed by A, B1, D, E, and F phylogroups. Several other features like the presence of virulent genes, ability to form biofilm, resistance to antibiotics, and toxin production were also checked [12]. In the present study, susceptibility of the 10 ocular *E. coli* isolates to 29 antibiotics belonging to 11 different classes was ascertained via phenotypic tests. Furthermore, whole-genome sequencing (WGS) of the 10 ocular surface *E. coli* isolates was accomplished and based on whole-genome SNP variation, the isolates were assigned to one or more of the nearest *E. coli* phylogenetic groups (A, B1, B2, C, D, E, and F) and to one or more of the eight pathotypes (EPEC, ETEC, EIEC, EAEC, DAEC, UPEC, STEC, or EHEC and ExPEC). Additional information on the presence of AMR genes, prophages, and genes involved in quorum sensing, biofilm formation, motility, and stress response, which contribute to AMR and pathogenicity, were also determined. This is the first report on the whole-genome analysis of 10 ocular *E. coli* strains. 

## 2. Materials and Methods 

### 2.1. Ethics 

This study was approved by the Ethics Committee of L V Prasad Eye Institute, Hyderabad, India (ECR/468/Inst./AP/2013, Ref No: LEC 03-15-029 (14th May 2018)).

### 2.2. Bacterial Strains and Antibiotic Susceptibility of the Ocular E. coli

The details of the patients from whom the 10 ocular strains of *E. coli* were isolated are given in Table S11. Two isolates were from the conjunctival swabs of two patients with conjunctivitis, two were from corneal scrapings of two patients with infectious keratitis, five were isolated from the vitreous fluid of five patients with endophthalmitis, and one from the pus of a patient with orbital cellulitis. The taxonomic characterization of the 10 ocular *E. coli* isolates was determined as described previously [18]. The 10 *E. coli* infections were community infections and presented with the infection when they visited the hospital. 

The susceptibility of the 10 isolates to 29 antibiotics (Table 1) was determined using the Vitek2 (BioMérieux, Paris, France) method and the serial dilution method [19,20,21]. The pattern of susceptibility was assigned as per European Committee on Antimicrobial Susceptibility Testing (EUCAST).

### 2.3. DNA Extraction and Whole-Genome Analysis

Genomic DNA was extracted from an overnight culture using a DNA isolation kit (Qiagen, Heilden, Germany), quality checked on 0.8% agarose gel run at 110 V for 30 min, and the concentration was determined using Qubit^®^ 2.0 Fluorometer [18].Whole-genome sequencing (WGS) was performed on the Illumina HiSeq 2500 platform at Xcelris Genomics Pvt. Ltd., Ahmedabad, India. Good quality genomic DNA (200 ng) was used for the preparation of the paired-end sequencing library using a Truseq Nano DNA Library Prep kit (Illumina, CA 92122, USA). 

### 2.4. Data Availability

All genome sequence data were submitted to the National Center for Biotechnology Information (NCBI) under Bioproject accession number PRJNA543974.

### 2.5. Assembly of Genomes

Genomes were assembled both by reference-based and de-novo-based assembly. In the former method, high-quality reads were mapped to a reference genome of *E. coli* strain K-12 substrain MG1655 (henceforth *E. coli* MG1655) genome (https://www.ncbi.nlm.nih.gov/nuccore/U00096.3/) [22] using BWA version 0.7.5a. Subsequently, SAM tools were used to call the consensus from the resultant BAM file. In de novo assembly, all 10 samples were de novo assembled separately using Velvet (v 1.2.10) assembler [19] at different k-mer ranges. Assemblies determined to be robust in terms of number of contigs, N50, and maximum scaffold length were selected for downstream analysis. The assembly elements were computed using in-house Perl script. The quality of the microbial genomes was based on the estimates of completeness and contamination, as monitored by CheckM [23]. 

### 2.6. Annotation of the Genomes and Pathway Analysis

BLAST v2.2.28+ was used for annotation of genes involved in antimicrobial resistance, virulence, prophages, motility, biofilm, quorum sensing, etc. The predicted proteins were also identified using the BLAST algorithm. Pathway analysis of the genes was performed using the KEGG automatic annotation server database using BLASTP with a default value of 60.

### 2.7. Non-Coding RNA Prediction

tRNAscan-SE v.1.31 [24,25] was used for identification of probable tRNA genes. These results were also validated using ARAGORN, a computer program which identifies tRNA and tmRNA (transfer-messenger RNA) genes. [26]. The location of ribosomal RNA genes was predicted using the Basic Rapid Ribosomal RNA Predictor.

### 2.8. Identification of Acquired Antimicrobial Resistance Genes, Plasmids, Virulence Genes, and Prophages

AMR genes including chromosomal mutations conferring AMR were detected using ResFinder 3.2 [27], plasmids using PlasmidFinder database (https://cge.cbs.dtu.dk/services/PlasmidFinder/version2.0) [28], and virulence genes using ABRicate (https://github.com/tseemann/abricate) and VFDB (Virulence Factor Data Base)database [29] as a reference. All the above genes were identified with a minimum coverage of 60% and minimum identity of 90%. Prophages in the genomes were identified using PHASTER (Phage Search Tool Enhanced Release) (http://phaster.ca/) [30,31]. Genes with 90% identity and 90% prophage sequence length coverage were considered for comparison of genomes.

### 2.9. SNP Detection and Phylogenetic Tree Construction

A whole-genome single nucleotide polymorphism (SNP)-based phylogenetic tree was constructed using kSNP3.0 software [32]. K-chooser was used to identify the optimal kmer length as 21 and to calculate the fraction of core kmers. We included genomes from all isolates and reference genomes belonging to phylogenetic groups A, B1, B2, C, D, E, and F. The parsimony tree was estimated based on SNP loci that occurred in at least 50% of the strains. Each strain was assigned to a phylogenetic group or a cryptic clade based on its position in the phylogenetic tree. In addition, Clermont typing was also used to determine the phylogroups of the ocular isolates [33,34], and the MLST 2.0 (Multi-Locus Sequence Typing 2.0) tool of Centre for Genomic Epidemiology was used to determine the sequence types (STs) of the ocular isolates [35].

### 2.10. Comparison of *E. coli* Genomes

BRIG (Blast Ring Image Generator) [36] was used to ascertain the similarities between a central reference sequence, the 10 ocular isolates, and reference sequences of *E. coli* phylogenetic groups A, B2, and C. L-2594/2017 was used as the reference sequence.

## 3. Results and Discussion

### 3.1. Whole-Genome Analysis and Antimicrobial Resistance Genes

Tyson et al. [37] and Moran et al. [38] suggested that a combination of whole-genome analysis (WGA) and phenotypic data would be most suitable to understand antimicrobial resistance. Accordingly the genomes of the 10 ocular strains were sequenced and compared with that of *E. coli* MG1655 with respect to several characteristics like genome coverage, completeness, the number of genes, the G+C% content of DNA, and number of tRNA genes (Table 1 and Table S1) which indicated a high degree of similarity and that majority of the genes were affiliated to *E. coli* MG1655. KEGG (Kyoto Encyclopedia of Genes and Genomes) pathway analysis also confirmed that majority of the genes were affiliated to *E. coli* and the mapped proteins represented metabolic pathways of major biomolecules and genes involved in genetic information processing, environmental information processing, cellular processes, drug resistance, etc. (Table S2). 

Resfinder 3.2. was used to identify 22 AMR genes conferring resistance to eight different classes of antibiotics (Table 2A) in the 10 ocular *E. coli* isolates. Isolate L-2594/2017 was the only isolate that possessed genes that conferred resistance to all the eight classes of antibiotics. Phenotypic studies were carried out to correlate the phenotypic susceptibility/resistance pattern to antibiotics with the absence or presence of AMR genes in the 10 isolates. When comparing the results in Table 2 and Table 3, certain generalizations emerged. The first was that in several cases, the presence of an AMR gene positively correlated with resistance to a particular antibiotic. For instance, resistance to one penicillin (ampicillin) and six cephalosporins (cefuroxime, cefuroxime axetil, ceftriaxone, cefepime, cefazolin, and ceftazidime) could be attributed to genes conferring resistance to penicillin (like *bla*_CTX-M-15_ and *dfrA17)* and to the six cephalosporins (like *aadA2*, *aadA5*, *bla*_OXA-1_, *bla*_NDM-5_, *dfrA12*, *dfrA17, bla*_CTX-M-15_, *bla*_TEM-1*B*_*, bla*_OXA-1_) that were detected (Table 2A and Table 3). Isolate L-1216/2010 was the most sensitive of all the 10 isolates and was resistant to only five antibiotics, namely nalidixic acid due to the presence of *bla*_TEM-1*B*_, and clindamycin and lincomycin due to the presence *of mdfA*, but resistant genes for minocycline and sulfanilamide were not detected, implying that resistance was due to some unknown genes (Table 2). It has been reported that *mdfA* also confers resistance to a broad range of antibiotics including tetracycline, chloramphenicol, erythromycin, daunomycin, puromycin, benzalkonium, rifampin, some aminoglycosides and fluoroquinolones, and organic cations such as ethidium bromide and tetraphenylphosphonium [39], and its presence in L-1216/2010 may therefore compensate for the absence of the specific genes conferring resistance to minocycline, a tetracycline antibiotic. It was also observed that sensitivity to an antibiotic could be attributed to the absence of a gene that confers resistance. For instance, all the isolates were susceptible to tigecycline, nitrofurantoin, and colistin, and they did not possess the corresponding resistant genes. In a recent study, Sun et al. [40] reported that tigecycline resistance was mediated by *tet(X4)* associated with IncQ1 plasmid in *E. coli*. Further the resistance to colistin, the polymixin antibiotic, could be attributed to the fact that lipid A modification, which is a prominent feature of polymyxin resistance, probably did not occur [41,42]. The second generalization was that the presence of an AMR gene may not result in a resistance phenotype if the gene is not expressed. For instance, L-1534/2017 was sensitive to ceftazidime although it possessed the AMR gene *bla*_TEM-1*B*_. We also observed resistance even in the absence of the AMR gene, implying that other undiscovered genes may be conferring resistance. In the ocular strains L-1534/2016 and L-3137/2017, we detected *bla*_TEM-1B_, which confers resistance to ampicillin. Intriguingly, seven other isolates did not possess *bla*_TEM-1B_ but were still resistant to ampicillin, indicating that another β-lactamase gene may be involved. This other β-lactamase gene is likely to be *bla*_CTX-M-15_ or *bla*_OXA-1_, which were detected in this study. A similar observation was also made with respect to susceptibility to fluoroquinolones. The AMR gene for fluoroquinolone is *aac(6’)-1b-cr*. Six isolates did not have this gene, but exhibited resistance to one or more of the six antibiotics tested, implying the existence of other genes conferring resistance to fluoroquinolones (Table 2 and Table 3). The additional possibility is that these organisms may have had chromosomal mutations conferring resistance to fluoroquinolones. Accordingly, we detected chromosomal mutations in*parE*, *gyrA*, and *parC* that conferred resistance to fluoroquinolones like nalidixic acid and ciprofloxacin (Table 2B).We observed one or more of these three generalizations across all the antibiotic groups studied (Table 2A,B and Table 3A). These results were in accordance with earlier results that demonstrated the presence of *bla*_CTX-M-15_ in *E. coli* strains from India [43,44] and from Europe, Asia, Canada, and United Kingdom [45,46,47,48]. We also confirmed that the plasmids harboring the *bla*_CTX-M-15_ gene were of the incompatibility group FII in 7 of the 10 isolates. We also detected the IncX3 plasmid, which plays an important role in the dissemination of *bla*_NDM-5_ [49]. The observed resistance to nalidixic acid could be attributable to the presence of *emrB*, which extrudes nalidixic acid. The *mphA* gene, which confers resistance to erythromycin and azithromycin, was also present in a few of the ocular isolates. Two of the three genes that confer resistance to sulfonamides, namely *sul1* and *sul2*, were also detected in a few of the ocular isolates. Studies on sulfonamide-resistant *Escherichia coli* from various animals and humans have indicated that *sul2* is the most prevalent, and *sul3* is restricted mainly to porcine *E. coli* isolates [50]. *Sul*-carrying plasmids belonged to diverse replicon types, and IncFII seems to be the dominant replicon type in *sul2*-carrying plasmids [51]. *dfrA* genes are known to confer resistance to trimethoprim, and their prevalence in *E. coli* isolates of human and animal origin has been well established [52,53]. The above results confirmed earlier studies pointing to a high concordance between the presence of genes conferring resistance to antibiotics and the phenotypic observation of antibiotic resistance. We compared the resistance profile of the ocular and urine isolates of *E. coli* from patients with ocular disease to different antimicrobial agents, and observed that both the ocular and urine isolates were more resistant to fluoroquinolones (73–90%) and cephalosporins (60–80%), but showed reduced resistance to aminoglycoside (10–23%), amphenicols (10–15%), and carbapenems (10–15%) (Table 3B). The urine isolates were more resistant than the ocular isolates to the antibiotics tested.

### 3.2. Plasmids and Virulence Genes

In this study, 11 different types of plasmid were identified (Table 4), with 7 affiliated to the Inc group, 3 to the Col group, and 1 to the repB plasmid group. Earlier studies have indicated that plasmids from Enterobacteriaceae contain antibiotic resistance genes [54,55,56,57], and a vast majority of these plasmids (>75%) can be classified into Inc groups or Rep types [54]. It is intriguing that *E. coli* L-1149/2016 was the only ocular strain without any plasmids, although it was resistant to 12 of the antibiotics tested (Table 1). In *E. coli* MG1655, we failed to detect any plasmids with either PlasmidFinder [28] or PlasmidSpades (https://github.com/ablab/spades) [20]. This study also confirmed that all the AMR genes were associated with a specific plasmid (Table S3), except that plasmid pT7-5, which harbors *mdfA*, was not detected. Furthermore, the STEC plasmid was not detected in the 10 ocular isolates (Table 5), confirming that STEC plasmid was rare in non-STEC strains of *E. coli* [21,58].

A total of 114 different virulent genes were detected across the 10 ocular isolates (Table 5) and 25 out of 114 were shared across all the isolates. Several virulent genes detected in this study were similar to those reported earlier [21], including aerobactin siderophore biosynthesis protein, cytotoxic necrotizing factor, secreted auto-transporter toxin, enterotoxin, yersiniabactin, enterobactin, aerobactin, adhesin, fimbriae, pilus, ABC transporter, type III secretion system effectors, hemoglobin proteases, serine protease, hemolysine genes, outer membrane protein genes, etc. (Table 5). It was observed that L-3003/2015 and L-2594/2017, which are affiliated to phylogenetic group A, possessed all the genes found in NA635, a group A reference strain, except *espY1*. At the same time, it was observed that *fimB* to *fimI* and *papB* to *papX* genes were present only in the ocular isolates (Tables S4 and S5). The majority of the virulent genes in the seven ocular isolates affiliated to phylogenetic group B2 were shared with the B2 reference strain CFT073. We also identified several *gspC* and *gspK* in these seven ocular isolates affiliated to phylogenetic group B2 *(*Table S4). We also compared the virulent genes of the 10 ocular isolates with type strains of the phylogenetic groups B1, B3, D, E, and F of *E. coli*, and the differences appeared to be more pronounced (Table S5).

### 3.3. Prophages in Ocular E. coli 

We report for the first time 24 prophages in 10 ocular *E. coli* (Table S6). Eleven out of the 24 prophages were unique and were detected only in a single isolate (Table S6). Enterobacteria phage lambda and Enterobacteria phage mEp460 were shared by eight isolates. Comparison of the prophages with other *E. coli* isolates indicated that four phages, namely Enterobacteria phage lambda, Enterobacteria phage mEp460, Enterobacteria phage P2, and Enterobacteria phage P4 were shared by all the groups of *E. coli* (ocular, EPEC, ETEC, ExPEC, UPEC, and environmental) in at least one strain, and five prophages, namely *Bacillus* phage *G*, Enterobacteria phage HK97, Enterobacteria phage P1, *Escherichia* phage *D108, Escherichia Stx1* converting phage, and *Escherichia* phage vB_EcoM-ep3 were unique to the ocular *E. coli* isolates (Table S7). We observed that the genome sizes of the 10 ocular isolates were larger than *E. coli* MG 1655 (Table 2), maybe due to the presence of prophages. This was in accordance with Perna et al. [59] and Hayashi et al. [60,61], who reported that the genomes of *E. coli* O157:H7 (strains EDL933 and RIMD0509952) were approximately 880 and 860 kb larger compared to *E. coli* MG1655, partly because these contained prophages and prophage-like elements. It was suggested that prophage sequences are beneficial since they confer AMR, help to overcome various types of stresses, and facilitate increase in growth and biofilm formation [62]. 

### 3.4. Genes Involved in Quorum Sensing, Biofilm Formation, and Motility in Ocular E. coli

Genes for quorum sensing (QS) (Table S8), genes facilitating biofilm formation (Table S9), and genes involved in motility (Table S10), which are important for AMR, were also identified. We have no direct proof of the involvement of these genes in AMR in ocular *E. coli*, but it is unequivocally established in the literature that QS biofilm formation [63,64,65] and motility [66,67,68,69] are linked to AMR. In a recent review, Rossi et al. [70] highlighted that motility is also downregulated in biofilm cells with simultaneous upregulation of adhesion factors [71]. In two earlier studies, we demonstrated biofilm formation potential in 7 of the above 10 ocular isolates, their increased resistance to antibiotics in the biofilm phase, identified genes that were overexpressed in the biofilm phase, and also identified five genes (*bdcR*, *mhpA*, *mhpB*, *ryfA*, and *tolA*) required for biofilm formation [18,72]. Studies have also implicated stress response regulators *Dgk*, sigma B, and *rpoS* in *Staphylococcus aureus* and in *E. coli* in biofilm formation [73,74,75,76]. Genes *spoT* and *relA* were present in 6 out of 10 ocular strains, and both these genes are involved in the stringent response in *E. coli* [77,78].

### 3.5. Phylogenetic Grouping of Ocular E. coli Based on BRIG

**Figure 1:**
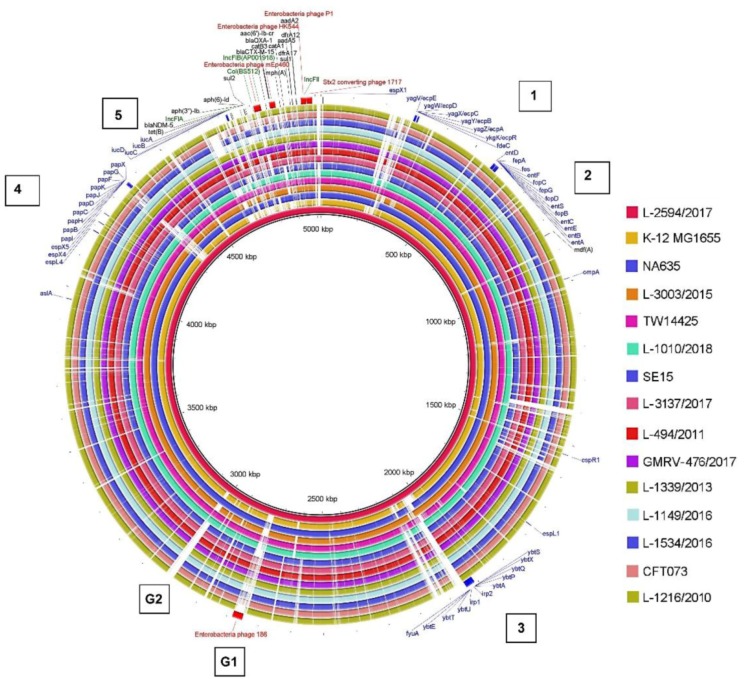
Comparison of ocular *E. coli* genomes and reference genomes of different phylogenetic groups. Ocular isolate L-2594/2017 was used as a reference. In the outermost ring, blue, black, red, and green indicate virulence genes, resistance genes, phages, and plasmids, respectively. Numerals 1 to 5 indicate the five clusters of virulent genes. BLASTn matches with less than 30% identity appear as blank spaces (gaps) in each ring. G1 and G2 indicate the gaps in the genomes. The image was prepared using Blast Ring Image Generator. Ring 1: Reference genome—L-2594/2017, Ring 2: *E. coli* MG1655, Ring 3: NA635, Ring 4: L-3003/2015, Ring 5: TW14425, Ring 6: L-1010/2018, Ring 7: SE15, Ring 8: L-3137/2017, Ring 9: L-494/2011, Ring 10: GMRV-476/2017, Ring 11: L-1339/2013, Ring 12: L-1149/2016, Ring 13: L-1534/2016, Ring 14: CFT073, and Ring 15: L-1216/2010.

BLAST Ring Image Generator (BRIG) was used to compare the whole genomes of the 10 ocular *E. coli* strains (Figure 1) with strains selected from phylogenetic groups A, B2, and C with respect to gaps in the genomes (like G1 and G2) indicative of the absence of a gene(s), presence/absence of virulence genes (blue), resistance genes (black), phages (red), and plasmids (green). Virulent genes (blue) were distributed across the genomes, and several of them were present as clusters (1 to 5). Clusters 1 and 2 were present in all the isolates and in the reference strains of phylogenetic groups A, B2 and C whereas genes in Clusters 3–5 were differentially distributed. It was also observed that L-3003/2015 (Ring 4) was very similar to phylogroup A reference strain NA635 (Ring 3), L-1010/2018 (Ring 6) to phylogroup C (Ring 5), and the remaining seven isolates (Rings 8-13 and15) were similar to phylogroup B2 (Rings 7 and 14). Perna et al. [59] and Hayashi et al. [60,61] previously reported that the genomes of pathogenic *E. coli* O157:H7 (strains EDL933 and RIMD0509952) were larger compared to *E. coli* MG1655, partly because of these prophages and prophage-like elements.

### 3.6. Phylogenetic Affiliation of the Ten Ocular E. coli Isolates with Other Phylogenomic Groups

This study raised the question as to the origin of the ocular *E. coli*. For this purpose, the genomes of the 10 ocular *E. coli* isolates were subjected to phylogroup identification using Clermont typing, which indicated that the 10 isolates were affiliated to three phylogenetic groups, A (L-2594/2017 and L-3003/2015), B2 (L-1339/2013, L-494/2011, L-3137/2017, L-1149/2016, L-1216/2010, L-1534/2016, and GMRV-476/2017), and C (L-1010/2018). Identical results were also obtained via phylogenetic analysis of whole-genome single nucleotide polymorphism (SNP) patterns using kSNP3.0 software [32] (data not shown). The tree also indicated that six strains were affiliated to ExPEC, two to EPEC, one to ETEC, and one to UPEC. These studies thus indicated a lack of concordance between the pathotype and phylogenetic group. Sequence typing also indicated that five isolates (L-1339/2013, L-494/2011, L-3137/2017, L-1149/2016, and GMRV-476/2017 ) were affiliated to ST131, and the remaining five were affiliated to ST10 (L-3003/2015), ST73 (L-1216/2010), ST410 (L-1010/2018), ST617 (L-2594/2017), and ST1193 (L-1534/2016), confirming a lack of concordance.). Our findings were partially in agreement with earlier studies indicating that extra-intestinal pathogenic *E. coli* are affiliated to phylogenetic groups A and B2 groups [32,79,80,81,82,83,84]. Phylogenetic groups B2 and D have been demonstrated to be linked to virulence determinants [17,85], antibiotic resistance [86,87,88], and possess virulence properties such as biofilm formation and hemolysin secretion when compared with phylogroup A and B1 isolates [87,89]. In this study, all the isolates, irrespective of their phylogenetic affiliations, had several AMR and virulent genes, biofilm genes, QS genes, etc., which facilitate their survival and pathogenic function. Further, it was observed as predicted that the 10 ocular *E. coli* isolates originated from fecal (EPEC and ETEC) or urine contamination (UPEC), or from extra-intestinal sources (ExPEC).

## 4. Conclusions

1. Whole-genome sequencing of 10 ocular strains of *E. coli* indicated increases in the genome length and the number of genes compared to a laboratory strain of *E. coli*.

2. Five of the 10 isolates were resistant to more than three classes of antibiotics, implying that these strains were MDR (Multi Drug Resistant) *E. coli.*

3. AMR genes were identified and a high concordance was observed between the presence of AMR genes and antibiotic resistance.

4. All the AMR genes were associated with a specific plasmid affiliated to the Inc group, the Col group, or the repB plasmid.

5. It was observed that the virulent genes *fimB* to *fimI*, *papB* to *papX*, and *gspC* and *gspK* genes were unique to the ocular isolates. 

6. Prophages, namely *Bacillus* phage *G*, Enterobacteria phage HK97, Enterobacteria phage P1, *Escherichia* phage *D108, Escherichia Stx1* converting phage, and *Escherichia* phage vB_EcoM-ep3 were also unique to the ocular *E. coli* isolates. 

7. Prophages and genes involved in quorum sensing and biofilm formation, which contribute to AMR and pathogenicity, were also detected in ocular *E. coli.*

8. The 10 ocular *E. coli* were affiliated to phylogenetic groups A (two isolates), B2 (seven isolates), and C (one isolate). 

9. The data also indicated that the 10 ocular *E. coli* isolates most likely originated from fecal (EPEC and ETEC), urine (UPEC), or from extra-intestinal sources (ExPEC), as per our prediction.

## Figures and Tables

**Table 1:** Characteristics of the whole-genome sequences of the 10 ocular *Escherichia coli* isolates.

Sl. No.	*E. coli* Strain	L-1339/ 2013	L-2594/ 2017	L-494/ 2011	L-3003/ 2015	L-1010/ 2018	L-3137/ 2017	L-1149/ 2016	GMRV-476/ 2017	L-1534/ 2016	L-1216/ 2010	K-12 MG1655
1	Sequencing data (GB)	0.83	0.83	1.63	1.87	1.04	1.09	0.88	1.03	1.02	0.88	
2	Completeness	99.97	99.97	99.97	99.97	99.62	99.97	99.97	99.97	99.91	99.97	99.97
3	Contamination	0.33	0.09	0.37	0.45	0.26	0.33	0.33	0.33	0.39	0.08	0.04
4	DNA coding sequence (%)	86.92	93.03	86.91	92.56	93.20	86.94	86.89	86.86	87.35	85.95	89
5	Genome length (kbp)	5351	5018	5420	4746	4864	5264	5119	5502	5046	5239	4639
6	Total number of tRNAs	85	87	85	88	87	85	84	85	85	84	87
7	G+C content of DNA (%)	50.6	50.7	50.7	50.7	50.6	50.7	50.7	50.6	50.6	50.5	50.8 *
8	Genes encoding a protein	5156	4816	5321	4467	4664	5074	4869	5402	4837	5008	4583 **

* Data were from https://www.pnas.org/content/103/34/12879.short#sec-8; ** Data from https://www.ncbi.nlm.nih.gov/Taxonomy/Browser/wwwtax.cgi? mode=Info & id=511145.

**Table:** (**A**)

S.No.	Class of Antibiotic	Resistance Gene	L-1339/ 2013	L-2594/ 2017	L-494/ 2011	L-3003/ 2015	L-1010/ 2018	L-3137/ 2017	L-1149/ 2016	GMRV-476/ 2017	L-1534/ 2016	L-1216/ 2010	Count
1	**Aminoglycoside**	*aph(3’’)-Ib*	-	+	-	-	-	-	-	-	+	-	2
		*aph(6)-Id*	-	+	-	-	-	-	-	-	+	-	2
		*aac(6’)-Ib-cr*	+	+	-	+	-	-	-	+	-	-	4
		*aadA5*	+	+	+	-	+	-	-	-	-	-	4
		*aac(3)-IIa*	+	-	+	-	-	-	-	-	-	-	2
		*aadA2*	-	+	-	-	-	-	-	-	-	-	1
2	**Amphenicol**	*catB3*	+	+	+	+	-	-	-	-	-	-	4
		*catA1*	-	+	-	-	-	-	-	-	-	-	1
3	**Beta-lactam**	*bla*_CTX-M-15_	+	+	+	+	+	+	+	+	-	-	8
		*bla*_NDM-5_	-	+	-	-	-	-	-	-	-	-	1
		*bla*_TEM-1*B*_	-	-	-	-	-	+	-	-	+	-	2
		*bla*_OXA-1_	+	+	+	+	-	-	-	+	-	-	5
4	**Fluoroquinolone**	*aac(6’)-Ib-cr*	+	+	-	+	-	-	-	+	-	-	4
5	**Lincosamide**	*mph(A)*	+	+	-	-	+	-	-	-	-	-	3
		*emrB*	-	-	-	+	-	-	-	-	-	-	1
		*mdfA*	-	+	+	+	+	+	+	+	+	+	9
6	**Sulfonamide**	*sul2*	-	+	-	-	-	-	-	-	+	-	2
		*sul1*	+	+	+	-	+	-	-	-	-	-	4
7	**Tetracycline**	*tet(B)*	-	+	-	-	-	-	-	-	-	-	1
		*tet(A)*	-	-	-	-	+	-	-	+	-	-	2
8	**Trimethoprim**	*dfrA17*	+	+	+	-	+	-	-	-	-	-	4
		*dfrA12*	-	+	-	-	-	-	-	-	-	-	1
Total		22	10	18	8	7	7	3	2	6	5	1	

* Genes were identified from the whole-genome sequence using ResFinder 3.2 (https://cge.cbs.dtu.dk/services/ResFinder/). (+) and (-) indicate presence and absence of gene, respectively.

**Table:** (**B**)

S. No.	*E. coli* Isolate	Chromosomal Mutation *		
		*parE*	*gyrA*	*parC*
1	L-1339/2013	I529L	S83L, D87N	S80I, E84V
2	L-2594/2017	S458A	S83L, D87N	S80I
3	L-494/2011	I529L	S83L, D87N	S80I, E84V
4	L-3003/2015	I529L	S83L, D87N	S80I, E84V
5	L-1010/2018	No mutation	S83L	G78D
6	L-3137/2017	S458A	S83L, D87N	S80I
7	L-1149/2016	S458A	S83L, D87N	S80I
8	GMRV-476/2017	L416F	S83L, D87N	S80I
9	L-1534/2016	I529L	S83L, D87N	S80I, E84V
10	L-1216/2010	I529L	S83L, D87N	S80I, E84V

* Chromosomal mutations were identified from the whole-genome sequence using ResFinder 3.2 (https://cge.cbs.dtu.dk/services/ResFinder/).

**Table:** (**A**)

Sl. No.	Antibiotic and Class		L-1339/ 2013 (CjS)	L-2594/ 2017 (V)	L-494/ 2011 (V)	L-3003/ 2015 (CrS)	L-1010/ 2018 (PC)	L-3137/ 2017 (V)	L-1149/ 2016 (CrS)	GMRV-476/ 2017 (V)	L-1534/ 2016 (CjS)	L-1216/ 2010 (V)	Resistance Genes
**1**	**Aminoglycosides**												
	**1**	Amikacin	R	S	S	S	S	S	S	S	S	S	*aac(6’)-Ib-cr*
	**2**	Gentamicin	R	S	R	S	S	S	S	S	S	S	*aadA5*, *aac(3)-Iia*
	**3**	Tobramycin	R	R	S	R	S	S	S	R	S	S	*aph(3’’)-Ib,aph(6)-Id,aac(6’)-Ib-cr,aadA5,aac(3)-IIa*
**2**	**Amphenicols**												
	**4**	Chloramphenicol	S	R	S	S	S	S	S	S	S	S	*catB*, *cat A1*
**3**	**β-lactam**												
	Penicillins												
	**5**	Ampicillin	R	R	R	R	R	R	R	R	R	S	*bla*_CTX-M-15_, *dfrA*17
	Cephalosporins												
	**6**	Cefuroxime	R	R	R	R	R	R	R	R	S	S	*aadA5*, *aadA2*, *dfrA12*, *dfrA17*, *bla*_NDM-5_, *bla*_OXA-1_
	**7**	Cefuroxime axetil	R	R	R	R	R	R	R	R	S	S	*aadA5*, *aadA2*, *dfrA12*, *dfrA17*, *bla*_NDM-5_, *bla*_OXA-1_
	**8**	Ceftriaxone	R	R	R	R	R	R	R	R	S	S	*bla*_TEM-1B_, *bla*_CTX-M-15_, *bla*_OXA-1_
	**9**	Cefepime	R	R	R	R	R	R	S	R	S	S	*bla*_TEM-1B_, *bla*_CTX-M-15_, *bla*_OXA-1_
	**10**	Cefazolin	R	R	R	R	R	R	R	R	R	S	*bla*_TEM-1B_
	**11**	Ceftazidime	R	R	R	R	R	R	R	R	S	S	*bla*_TEM-1B_
**3**	Carbapenems												
	**12**	Ertapenem	S	R	S	S	S	S	S	S	S	S	*bla*_CTX-M-15_, *bla*_NDM-5_, *bla*_OXA-1_
	**13**	Imipenem	S	R	S	S	S	S	S	S	S	S	*bla*_NDM-5_, *bla*_OXA-1_
	**14**	Meropenem	S	R	S	S	S	S	S	S	S	S	*bla*_NDM-5_, *bla*_OXA-1_
**4**	**Fluoroquinolones**												
	**15**	Gatifloxacin	R	R	R	R	R	R	R	R	R	S	
	**16**	Moxifloxacin	R	R	R	R	R	R	R	R	R	S	
	**17**	Ciprofloxacin	R	R	R	R	R	S	R	R	R	S	*aac(6’)-Ib-cr*
	**18**	Ofloxacin	R	R	R	R	R	R	R	R	R	S	
	**19**	Norfloxacin	R	R	R	R	R	R	R	R	R	S	*cat A1*
	**20**	Nalidixic Acid	R	R	R	R	R	R	R	R	R	R	*bla*_TEM-1B_
**5**	**Glycylcyclines**												
	**21**	Tigecycline	S	S	S	S	S	S	S	S	S	S	
**6**	**Nitrofurans**												
	**22**	Nitrofurantoin	S	S	S	S	S	S	S	S	S	S	
**7**	**Polymyxins**												
	**23**	Colistin	S	S	S	S	S	S	S	S	S	S	
**8**	**Tetracycline**												
	**24**	Doxycycline	S	R	S	S	R	R	R	R	R	S	*tet(A)*, *tet(B)*
	**25**	Minocycline	S	S	S	S	S	S	S	S	S	R	*tet(A)*, *tet(B)*
**9**	**Lincosamide**												
	**26**	Clindamycin	R	R	R	R	R	R	R	R	R	R	*mph(A)*, *emrB*, *mdfA*
	**27**	Lincomycin	R	R	R	R	R	R	R	R	R	R	*mph(A)*, *emrB*, *mdfA*
**10**	**Sulfanilamide**												
	**28**	Sulfanilamide	R	R	R	R	R	R	R	R	R	R	*sul1*, *sul2*
**11**	**Trimethoprim**												
	**29**	Trimethoprim	R	R	R	R	S	S	S	S	R	S	*dfrA17*, *dfrA12*
Resistant (29 antibiotics tested)			20	23	18	18	17	16	16	18	13	5	
Resistant to number of classes			5	5	6	5	4	4	4	5	5	3	

* Sample: CjS: conjunctival swab; V; vitreous; CrS: corneal scraping; PC: pus-orbital cellulitius. S: Sensitive; R: resistant.

**Table:** (**B**)

Antibiotic	Antibiotics (Number of Urine Samples Tested)	Resistance in Urine Isolates (%)	Resistance in Ocular Isolates (%)
Aminoglycoside	Gentamycin (26)	23.08	20
	Amikacin (86)	16.28	10
Amphenicols	Chloramphenicol (86)	15.12	10
Cephalosporins	Ceftazidime (84)	66.67	80
	Cefuroxime (25)	60.00	80
Carbapenems	Imipenem (67)	15.38	10
Fluoroquinolones	Ciprofloxacin (86)	82.56	80
	Ofloxacin (86)	75.58	90
	Moxifloxacin (86)	77.91	90
	Gatifloxacin (86)	73.26	90
	Norfloxacin (67)	79.10	90

**Table 4:** Plasmids in the 10 ocular *E. coli* * isolates.

Sl. No.	Plasmid	L-1339/2013	L-2594/2017	L-494/2011	L-3003/2015	L-1010/2018	L-3137/2017	L-1149/2016	GMRV-476/2017	L-1534/2016	L-1216/2010	Count
**1**	*Col(BS512)*	-	+	-	-	-	-	-	+	+	-	3
**2**	*Col(MG828)*	-	-	-	-	-	+	-	-	-	-	1
**3**	*Col156*	-	-	-	-	+	+	-	-	+	+	4
**4**	*IncFIA*	+	+	+	+	+	-	-	+	+	-	7
**5**	*IncFIB(AP001918)*	+	+	+	+	+	+	-	+	+	+	9
**6**	*IncFII*	+	+	+	+	+	+	-	+	-	-	7
**7**	*IncFII(29)*	-	-	-	-	-	-	-	-	-	+	1
**8**	*IncQ1*	-	-	-	-	-	-	-	-	+	-	1
**9**	*IncX4*	+	-	-	-	-	-	-	-	-	-	1
**10**	*IncY*	-	-	+	-	-	-	-	-	-	-	1
**11**	*p0111/repB*	-	-	-	-	-	-	-	+	-	-	1
**Total**		4	4	4	3	4	4	0	5	5	3	

* Plasmids were detected using PlasmidFinder 2.1 (https://cge.cbs.dtu.dk/services/Plasmid Finder/). (+) and (-) indicate presence and absence of plasmid respectively.

**Table 5:** Virulence genes * in 10 ocular *E. coli* isolates.

Sl. No.	Protein and Gene		Plasmid	L-1339/ 2013	L-2594/ 2017	L-494/ 2011	L-3003/ 2015	L-1010/ 2018	L-1037/ 2017	L-1149/ 2016	GMRV-457/ 2017	L-1534/ 2016	L-1216/ 2010	Count
1	Adhesin	*fdeC*	pITS 24	+	+	+	+	+	+	+	+	+	+	10
2	Secreted auto transpoter toxin	*Sat*	55989p	+	-	+	-	-	+	+	+	+	-	6
3	Aerobactin receptor	*iutA*	pAPEC-O2-ColV	-	-	-	-	-	-	-	+	-	-	1
4	Aerobactin siderophore biosynthesis protein	*iucA*, *iucB*, *iucC*	pO83_CORR	+	+	+	+	+	+	+	+	+	-	9
5	A fimbrial adhesin	*afaA*, *afaB-I*, *afaC-I*, *afaD*, *afaE-I*	pILL1101, pO86A1	-	-	-	-	+	-	-	-	-	-	1
6	polysaccharide export ATP-binding protein	*kpsT*, *kpsD*, *kpsM*	pCP53-92k, pSR128	+	-	+	-	-	+	+	+	+	+	7
7	Cytotoxic necrotizing factor 1	*cnf1*	pVir68	-	-	-	-	-	-	+	-	-	+	2
8	Daaf protein	*daaF*	-	-	-	-	-	+	-	-	-	-	-	1
9	Dradhesins	*draP*	-	-	-	-	-	+	-	-	-	-	-	1
10	*E. coli* common pilus chaperone	*yagV/ecpE*, *yagX/ecpC*, *yagY/ecpB*, *yagZ/ecpA*	-	+	+	+	-	+	+	+	+	+	+	9
11	Enterobactin synthase multienzyme complex	*entA*, *entB*, *entC*, *entD*, *entE*, *entF*, *entS*	pITS 12, pITS 1, pITS 24, pITS 31, pITS 21	+	+	+	+	+	+	+	+	+	+	10
12	Enterobactin/ferric enterobactin esterase	*Fes*	pAPEC-1	+	+	+	+	+	+	+	+	+	+	10
13	Enterotoxin	*senB*	pEC14_114	-	-	-	-	+	+	-	-	+	+	4
14	F1C major fimbrial subunit precursor	*focA*, *focF*, *focG*, *focH*	pPKL143	-	-	-	-	-	-	-	-	-	+	1
15	Ferrienterobactin ABC transporter permease	*fepA*, *fepB*, *fepC*, *fepD*, *fepG*	pITS24, pTBnb2, pAPEC-O103-ColBM	+	+	+	+	+	+	+	+	+	+	10
16	Fimbrial switch regulatory protein	*sfaB*, *sfaC*, *sfaF*	-	-	-	-	-	-	-	-	-	-	+	1
17	General secretion pathway protein C, D, E, L, F, G, H, I, J, K, M	*gspC*, *gspD*, *gspE*, *gspL*, *gspF*, *gspG*, *gspH*, *gspI*, *gspJ*, *gspK*, *gspM*	p14EC047c, p2009C-4207, pMRE600-1, p2009C-4207	-	-	+	-	-	+	+	-	+	+	5
18	Glucosyltransferase	*iroB*, *iroC*, *iroE*, *iroD*, *iroN*	p2HS-C-2,pSA186_2	-	-	-	-	-	-	-	-	-	+	1
19	Haemoglobin protease	*Vat*	-	-	-	-	-	-	-	-	-	+	+	2
20	Hemolysin A, B, C, D	*hlyA*, *hlyB*, *hlyC*, *hlyD*	pO157, pO158	-	-	+	-	-	+	+	-	-	+	4
21	Outer membrane protein	*ompA*	pO113	+	+	+	+	+	+	+	+	+	+	10
22	Pesticin/yersiniabactin receptor protein	*fyuA*	-	+	+	+	+	+	+	+	+	+	+	10
23	Pilus assembly protein	*papB*, *papD*, *papC*, *papI*, *papX*, *papF*, *papG*, *papH*, *papJ*, *papK*	pDD3, pDD4, p600468_158, p503829_52, p300709_60,	+	+	+	-	-	+	+	+	-	+	7
24	Polymerized tip adhesin of ECP fibers	*yagW/ecpD*	pVir68	+	+	+	-	+	+	+	+	+	+	9
25	Putative arylsulfatase	*aslA*	p5C10	+	+	+	+	-	+	+	+	+	+	9
26	Putative heme-binding protein	*chuA*, *chuS*, *chuT*, *chuU*, *chuV*, *chuW*, *chuX*, *chuY*	pYUK1	+	-	+	-	-	+	+	+	+	+	7
27	Regulator protein ecpr	*ykgK/ecpR*	-	+	+	+	-	+	+	+	+	+	+	9
28	Serine protease precursor	*Pic*	pHUSEC41-2	-	-	-	-	-	-	-	-	-	+	1
29	Tir-domain-containing protein	*tcpC*	pNB10	-	-	-	-	-	-	-	-	-	+	1
30	Fimbriae regulatory protein	*fimA*, *fimB*, *fimC*, *fimD*, *fimE*, *fimF*, *fimG*, *fimH*, *fimI*	pSLD203, pWRS17-4Z7, pfimF, pVir68, pORN160, pWRS1-17, pORN310, pEsST410_NW_4	+	-	+	+	+	+	+	+	+	+	9
31	Type III secretion system effector	*espL1*, *espL4*, *espX1*, *espX4*, *espX5*	pO157	-	+	-	+	+	-	-	-	-	-	3
32	Yersiniabactin protein	*irp1*, *irp2*	pUC18	+	+	+	+	+	+	+	+	+	+	10
33	Yersiniabactin transcriptional regulator	*ybtA*, *ybtE*, *ybtP*, *ybtQ*, *ybtS*, *ybtT*, *ybtU*, *ybtX*	pET3c-YbtA, pCP1, pPSN345, pEUYbtP, pSDR498.1	+	+	+	+	+	+	+	+	+	+	10
Total				18	15	20	12	18	21	21	19	20	26	

* Abricate virulence finder database (https://github.com/tseemann/abricate) was used for the presence (+) or absence (-) of a gene.

## Supplementary Materials

The following are available online at https://www.mdpi.com/2076-2607/8/3/422/s1, Table S1: Identification of probable tRNA genes* in the 10 ocular *E. coli* isolates. Table S2: Enrichment of genes* in the 10 ocular *E. coli* isolates. Table S3: Antimicrobial-resistant genes associated with the plasmids in the 10 ocular *E. coli* isolates. Table S4: Comparison of virulent genes in the 10 ocular *E. coli* isolates and *E. coli* type strains associated with phylogenomic groups A, B2, and C. Table S5: Comparison of virulent genes in the 10 ocular *E. coli* isolates and *E. coli* type strains associated with phylogenomic groups A to F. Table S6: Comparison of prophages in ocular, EPEC, ETEC, ExPEC, UPEC, and environmental *E. coli* strains. Table S7: Characteristics of patients from whom the 10 ocular *E. coli* isolates were collected. Table S8: Number of quorum sensing genes * in ocular *E. coli* isolates. Table S9: Biofilm genes * in ocular *E. coli* isolates. Table S10: Motility genes * in ocular *E. coli* isolates. Table S11: Characteristics of patients from whom the 10 ocular *E. coli* were isolated.
