# Supplementary material for: Phylogenetic Grouping of Human Ocular Escherichia coli Based on Whole-Genome Sequence Analysis
Source: Microorganisms. 2020 Mar 17;8(3):422. doi: 10.3390/microorganisms8030422 (PMC7143957; doi:10.3390/microorganisms8030422)
Supplement: Supplementary file 1 [file microorganisms-08-00422-s001.pdf]

## SUPPLEMENTARY TABLES

**Table S1.** Identification of probable tRNA genes* in the 10 ocular *E. coli* isolates

| Sl. No | *E. coli* strain | tRNAs decoding standard 20 amino acids | Seleno-cysteine tRNAs | Possible suppressor tRNAs (CTA,TTA, TCA) | tRNAs with undetermined /unknown isotypes | tRNAs with mis-match isotypes | Predicted pseudo-genes | Total tRNAs by tRNA-scan-SE | Total tRNAs by ARAGORN |
|---|---|---|---|---|---|---|---|---|---|
| **1** | **L-1339/2013** | 83 | 1 | 0 | 1 | 0 | 0 | 85 | 86 |
| **2** | **L-2594/2017** | 85 | 1 | 0 | 0 | 0 | 1 | 87 | 88 |
| **3** | **L-494/2011** | 83 | 1 | 0 | 1 | 0 | 0 | 85 | 86 |
| **4** | **L-3003/2015** | 85 | 1 | 0 | 1 | 0 | 1 | 88 | 87 |
| **5** | **L-1010/2018** | 84 | 1 | 0 | 2 | 0 | 0 | 87 | 88 |
| **6** | **L-3137/2017** | 83 | 1 | 0 | 1 | 0 | 0 | 85 | 85 |
| **7** | **L-1149/2016** | 82 | 1 | 0 | 1 | 0 | 0 | 84 | 85 |
| **8** | **GMRV-476/2017** | 83 | 1 | 0 | 1 | 0 | 0 | 85 | 86 |
| **9** | **L-1534/2016** | 83 | 1 | 0 | 1 | 0 | 0 | 85 | 85 |
| **10** | **L-1216/2010** | 82 | 1 | 0 | 1 | 0 | 0 | 84 | 84 |
| **11** | **K-12 MG1655** | 86 | 1 | 0 | 1 | 0 | 1 | 89 | NA |

* The number tRNAs genes were detected using using tRNAscan-SE and ARAGORN.

**Table S2.** Enrichment of genes * in the 10 ocular *E. coli* isolates

| Sl. No. | KEGG Category* | L-1339/ 2013 | L-2594/ 2017 | L-494/ 2011 | L-3003/ 2015 | L-1010/ 2018 | L-3137/ 2017 | L-1149/ 2016 | GMRV-476/ 2017 | L-1534/ 2016 | L-1216/ 2010 |
|---|---|---|---|---|---|---|---|---|---|---|---|
| **1** | 09101 Carbohydrate metabolism | 505 | 487 | 501 | 482 | 504 | 501 | 503 | 503 | 500 | 496 |
| **2** | 09102 Energy metabolism | 184 | 184 | 184 | 181 | 184 | 184 | 184 | 184 | 182 | 182 |
| **3** | 09103 Lipid metabolism | 87 | 89 | 87 | 89 | 87 | 87 | 87 | 87 | 87 | 93 |
| **4** | 09104 Nucleotide metabolism | 154 | 157 | 154 | 152 | 157 | 154 | 154 | 154 | 154 | 154 |
| **5** | 09105 Amino acid metabolism | 256 | 275 | 254 | 272 | 277 | 254 | 256 | 256 | 243 | 248 |
| **6** | 09106 Metabolism of other amino acids | 84 | 80 | 78 | 80 | 83 | 78 | 83 | 82 | 80 | 81 |
| **7** | 09107 Glycan metabolism | 60 | 60 | 60 | 60 | 58 | 60 | 60 | 60 | 57 | 57 |
| **8** | 09108 Metabolism of cofactors and vitamins | 185 | 186 | 185 | 185 | 185 | 185 | 185 | 185 | 185 | 188 |
| **9** | 09109 Metabolism of terpenoids and polyketides | 39 | 36 | 39 | 36 | 34 | 39 | 39 | 39 | 37 | 34 |
| **10** | 09110 Biosynthesis of secondary metabolites | 46 | 45 | 46 | 43 | 42 | 46 | 46 | 46 | 46 | 43 |
| **11** | 09111 Xenobiotics metabolism | 74 | 82 | 74 | 79 | 79 | 74 | 74 | 74 | 62 | 66 |
| **12** | 09121 Transcription | 4 | 4 | 4 | 4 | 4 | 4 | 4 | 4 | 4 | 4 |
| **13** | 09122 Translation | 86 | 86 | 86 | 85 | 86 | 86 | 86 | 86 | 86 | 86 |
| **14** | 09123 Folding, sorting and degradation | 54 | 53 | 54 | 54 | 53 | 54 | 54 | 54 | 54 | 54 |
| **15** | 09124 Replication and repair | 89 | 91 | 95 | 91 | 91 | 92 | 89 | 92 | 91 | 91 |
| **16** | 09131 Membrane transport | 268 | 253 | 278 | 251 | 259 | 276 | 276 | 269 | 268 | 276 |
| **17** | 09132 Signal transduction | 164 | 167 | 164 | 168 | 169 | 164 | 164 | 164 | 165 | 167 |
| **18** | 09141 Transport and catabolism | 8 | 8 | 8 | 8 | 8 | 8 | 8 | 8 | 8 | 8 |
| **19** | 09142 Cell motility | 58 | 44 | 58 | 60 | 60 | 58 | 58 | 58 | 59 | 58 |
| **20** | 09143 Cell growth and death | 20 | 19 | 20 | 19 | 19 | 20 | 20 | 20 | 19 | 19 |
| **21** | 09145 Cellular community prokaryotes | 153 | 159 | 156 | 160 | 159 | 156 | 158 | 152 | 157 | 159 |
| **22** | 09149 Aging | 14 | 14 | 14 | 14 | 14 | 14 | 14 | 14 | 14 | 14 |
| **23** | 09151 Immune system | 6 | 6 | 6 | 6 | 6 | 6 | 6 | 6 | 6 | 6 |

| Sl. No. | KEGG Category* | L-1339/ 2013 | L-2594/ 2017 | L-494/ 2011 | L-3003/ 2015 | L-1010/ 2018 | L-3137/ 2017 | L-1149/ 2016 | GMRV-476/ 2017 | L-1534/ 2016 | L-1216/ 2010 |
|---|---|---|---|---|---|---|---|---|---|---|---|
| **24** | 09152 Endocrine system | 16 | 16 | 16 | 16 | 16 | 16 | 16 | 16 | 16 | 16 |
| **25** | 09154 Digestive system | 2 | 2 | 2 | 2 | 2 | 2 | 2 | 2 | 2 | 2 |
| **26** | 09155 Excretory system | 2 | 2 | 2 | 2 | 2 | 2 | 2 | 2 | 2 | 2 |
| **27** | 09156 Nervous system | 10 | 7 | 7 | 7 | 8 | 7 | 9 | 9 | 8 | 8 |
| **28** | 09159 Environmental adaptation | 6 | 6 | 6 | 6 | 6 | 6 | 6 | 6 | 6 | 6 |
| **29** | 09161 Cancers: Overview | 24 | 23 | 25 | 23 | 23 | 25 | 25 | 24 | 23 | 23 |
| **30** | 09162 Cancers: Specific types | 6 | 6 | 6 | 6 | 6 | 6 | 6 | 6 | 6 | 6 |
| **31** | 09163 Immune diseases | 2 | 2 | 2 | 2 | 2 | 2 | 2 | 2 | 2 | 2 |
| **32** | 09164 Neurodegenerative diseases | 8 | 8 | 8 | 8 | 8 | 8 | 8 | 8 | 8 | 8 |
| **33** | 09166 Cardiovascular diseases | 6 | 6 | 6 | 6 | 6 | 6 | 6 | 6 | 6 | 6 |
| **34** | 09167 Endocrine and metabolic diseases | 11 | 8 | 8 | 8 | 9 | 8 | 10 | 10 | 9 | 9 |
| **35** | 09171 Infectious diseases: Bacterial | 32 | 26 | 34 | 30 | 34 | 34 | 35 | 32 | 31 | 34 |
| **36** | 09172 Infectious diseases: Viral | 2 | 2 | 2 | 2 | 2 | 2 | 2 | 2 | 2 | 2 |
| **37** | 09174 Infectious diseases: Parasitic | 2 | 2 | 2 | 2 | 2 | 2 | 2 | 2 | 2 | 2 |
| **38** | 09175 Drug resistance: Antimicrobial | 64 | 60 | 64 | 60 | 60 | 64 | 64 | 64 | 64 | 64 |
| **39** | 09176 Drug resistance: Antineoplastic | 9 | 9 | 9 | 9 | 9 | 9 | 9 | 9 | 9 | 9 |
| **40** | 09181 Protein families: metabolism | 326 | 326 | 327 | 324 | 325 | 327 | 327 | 326 | 318 | 321 |
| **41** | 09182 Protein families: genetic information processing | 742 | 753 | 748 | 750 | 755 | 744 | 738 | 745 | 739 | 738 |
| **42** | 09183 Protein families: signaling and cellular processes | 939 | 912 | 950 | 910 | 946 | 945 | 944 | 941 | 934 | 949 |

*, The numbers indicate the no. of genes as detected by KEGG pathway analysis (https://www.genome.jp/kegg/pathway.html).

**Table S3.** Antimicrobial resistant genes associated with the plasmids in the 10 ocular *E. coli* isolates

| Plasmid | Antimicrobial resistant genes |
| --- | --- |
| IncFIA | *bla*$_{TEM-1B}$, *mph(A)*, *aadA5*, *sul2*, *sul1*, *tetB*, *tetA*, *dfrA17*, *bla*$_{CTX-M-15}$, *bla*$_{OXA-1,}$ *catB3*, *emrB*, *aph(3'')-Ib*, *aac(3)-IIa*, *aph(6)-Id* |
| IncFIB (AP001918) | *bla*$_{TEM-1B}$, *mph(A)*, *aadA2*, *aadA5*, *sul2*, *sul1*, *tetB*, *tetA*, *dfrA17*, *dfrA12*, *aac(6')-Ib-cr*, *bla*$_{OXA-1,}$ *catB3*, *emrB*, *aph(3'')-Ib*, *aac(3)-IIa*, *aph(6)-Id* |
| IncFII | *bla*$_{TEM-1B}$, *catA1*, *aadA2*, *sul2*, *sul1*, *tetB*, *tetA*, *dfrA12*, *bla*$_{CTX-M-15}$, *bla*$_{OXA-1,}$ *catB3*, *bla*$_{NDM-5,}$ *emrB*, *aph(3'')-Ib*, *aac(3)-IIa*, *aph(6)-Id* |
| IncFII(29) | *bla*$_{TEM-1B}$, *aadA2*, *sul2*, *tetA*, *tetB*, *dfrA12* |
| IncQ1 | *catA1*, *sul1*, *sul2*, *bla*$_{TEM-1B}$, *tetA*, *aac(6')-Ib-cr*, |
| IncX4 | |
| IncY | *sul2*, *tetA* |
| Col156 | *sul2*, *tetA* |
| Col(BS512) | *aadA5*, *sul2*, *sul1*, *tetB*, *tetA* |
| Col(MG828) | *aadA5*, *sul1*, *bla*$_{TEM-1B}$, *tetA* |
| p0111/repB | *aadA2*, *bla*$_{TEM-1B}$, *dfrA17*, *aadA5*, *sul2*, *dfrA12* |
| pT7-5 | *mdfA* |

**Table S4.** Comparison of virulent genes in the 10 ocular *E. coli* isolates and *E. coli* type strains associated with phylogenomic groups A, B2 and C

| Sl. No. | Gene | A | | | | B2 | | | | | | | | | C | | Count |
|---|---|---|---|---|---|---|---|---|---|---|---|---|---|---|---|---|---|
| | | L-2594/2017 | L-3003/2015 | NA635* | k-12 MG1655* | L-1216/2010 | L-1534/2016 | L-1149/2016 | L-1339/2013 | GMRV-476/2017 | L-494/2011 | L-3137/2017 | CFT073 | SE15* | L-1010/2018 | TW14425* | |
| 1 | afaA, afaB-I, afaC-I, afaD, afaE-I | - | - | - | - | - | - | - | - | - | - | - | - | - | + | - | 1 |
| 2 | aslA | + | + | + | + | + | + | + | + | + | + | + | + | + | - | - | 13 |
| 3 | cfaA, cfaD | - | - | - | - | - | - | - | - | - | - | - | - | - | - | + | 1 |
| 4 | chuA, chuS, chuT, chuU, chuV,chuW, chuX, chuY | - | - | - | - | + | + | + | + | + | + | + | + | + | - | - | 9 |
| 5 | cnf1 | - | - | - | - | + | - | + | - | - | - | - | - | - | - | - | 2 |
| 6 | csaC, csaE | - | - | - | - | - | - | - | - | - | - | - | - | - | - | + | 1 |
| 7 | daaF | - | - | - | - | - | - | - | - | - | - | - | - | - | + | - | 1 |
| 8 | draP | - | - | - | - | - | - | - | - | - | - | - | - | - | + | - | 1 |
| 9 | entA, entB, entC, entE, entF, entS | + | + | + | + | + | + | + | + | + | + | + | + | + | + | + | 15 |
| 10 | entD | + | + | + | + | + | - | - | - | - | - | - | + | - | + | + | 8 |
| 11 | espL1, espX4 espX5 | + | + | + | + | - | - | - | - | - | - | - | - | - | + | + | 6 |
| 12 | espL4 | + | + | + | + | - | - | - | - | - | - | - | - | - | - | - | 4 |
| 13 | espX1 | + | + | + | - | - | - | - | - | - | - | - | - | - | + | + | 5 |
| 14 | espY1 | - | - | + | - | - | - | - | - | - | - | - | - | - | - | - | 1 |

| Sl. No. | Gene | A | | | | B2 | | | | | | | | | C | | |
|---|---|---|---|---|---|---|---|---|---|---|---|---|---|---|---|---|---|
| | | L-2594/2017 | L-3003/2015 | NA635* | k-12 MG1655* | L-1216/2010 | L-1534/2016 | L-1149/2016 | L-1339/2013 | GMRV-476/2017 | L-494/2011 | L-3137/2017 | CFT073 | SE15* | L-1010/2018 | TW14425* | Count |
| 15 | etpB | - | - | - | - | - | - | - | - | - | - | - | - | - | - | + | 1 |
| 16 | fdeC | + | + | + | - | + | + | + | + | + | + | + | + | + | + | + | 14 |
| 17 | fepA, fepB , fepC, fepD , fepG, fes | + | + | + | + | + | + | + | + | + | + | + | + | + | + | + | 15 |
| 18 | fimA | - | - | - | + | + | + | + | + | + | + | + | + | + | + | - | 11 |
| 19 | fimB | - | + | - | + | + | + | - | - | - | + | + | + | + | + | - | 9 |
| 20 | fimC, fimD fimE, fimF, fimG, fimH, fimI | - | + | - | + | + | + | + | + | + | + | + | + | + | + | - | 12 |
| 21 | focA, focC ,focD, focF ,focG, focH | - | - | - | - | + | - | - | - | - | - | - | + | - | - | - | 2 |
| 22 | fyuA | + | + | + | - | + | + | + | + | + | + | + | + | + | + | - | 13 |
| 23 | gspC | - | - | - | + | + | + | + | - | - | + | + | - | + | - | + | 8 |
| 24 | gspD, gspE gspF, gspG ,gspH, gspI, gspJ, | - | - | - | - | + | + | + | - | - | + | + | - | + | - | + | 7 |
| 25 | gspK | - | - | - | - | + | + | + | - | - | + | + | - | + | + | + | 8 |
| 26 | gspL, gspM | - | - | - | + | + | + | + | + | + | + | + | + | + | + | + | 12 |
| 27 | hlyA, hlyB , hlyC, hlyD | - | - | - | - | + | - | + | - | - | + | + | + | - | - | - | 5 |
| 28 | iroB, iroC , iroD, iroE ,iroN, | - | - | - | - | + | - | - | - | - | - | - | + | - | - | - | 2 |

| Sl. No. | Gene | A | | | | B2 | | | | | | | | | C | | |
|---|---|---|---|---|---|---|---|---|---|---|---|---|---|---|---|---|---|
| | | L-2594/2017 | L-3003/2015 | NA635* | k-12 MG1655* | L-1216/2010 | L-1534/2016 | L-1149/2016 | L-1339/2013 | GMRV-476/2017 | L-494/2011 | L-3137/2017 | CFT073 | SE15* | L-1010/2018 | TW14425* | Count |
| 29 | irp1 | + | + | + | - | + | + | + | + | + | + | + | + | + | + | - | 13 |
| 30 | irp2 | + | + | + | - | + | + | + | + | + | + | + | - | + | + | - | 12 |
| 31 | iucA, iucB , iucD | + | + | + | - | - | + | + | + | + | + | + | + | - | + | - | 11 |
| 32 | iucC | + | + | + | - | - | + | + | + | - | - | + | + | - | + | - | 9 |
| 33 | iutA | - | - | - | - | - | - | - | - | - | - | - | + | - | - | - | 1 |
| 34 | kpsD, kpsM | - | - | - | - | + | + | + | + | + | + | + | + | + | - | - | 9 |
| 35 | kpsT | - | - | - | - | - | + | - | - | - | - | - | - | - | - | - | 1 |
| 36 | ompA | + | + | + | + | + | + | + | + | + | + | + | + | + | + | + | 15 |
| 37 | papA, papE | - | - | - | - | - | - | - | - | - | - | - | + | - | - | - | 1 |
| 38 | papB | + | - | - | - | + | + | - | - | + | + | + | + | - | - | - | 7 |
| 39 | papC, papD, papF, papG | + | - | - | - | + | - | + | + | + | + | + | + | - | - | - | 8 |
| 40 | papH | - | - | - | - | + | - | - | - | - | - | - | + | - | - | - | 2 |
| 41 | papI | + | - | - | - | + | + | + | + | + | + | + | + | - | - | - | 9 |
| 42 | papJ | + | - | - | - | + | - | + | + | + | + | + | + | - | - | - | 8 |
| 43 | papK | + | - | - | - | + | - | + | + | - | + | + | + | - | - | - | 7 |
| 44 | papX | + | - | - | - | - | + | + | + | + | + | + | + | - | - | - | 8 |

| Sl. No. | Gene | A | | | | B2 | | | | | | | | | C | | Count |
|---|---|---|---|---|---|---|---|---|---|---|---|---|---|---|---|---|---|
| | | L-2594/2017 | L-3003/2015 | NA635* | k-12 MG1655* | L-1216/2010 | L-1534/2016 | L-1149/2016 | L-1339/2013 | GMRV-476/2017 | L-494/2011 | L-3137/2017 | CFT073 | SE15* | L-1010/2018 | TW14425* | |
| 45 | Pic | - | - | - | - | + | - | - | - | - | - | - | + | - | - | - | 2 |
| 46 | Sat | - | - | - | - | - | + | + | + | + | + | + | + | - | - | - | 7 |
| 47 | senB | - | - | - | - | + | + | - | - | - | - | + | - | + | + | - | 5 |
| 48 | sfaB, sfaC | - | - | - | - | + | - | - | - | - | - | - | + | - | - | - | 2 |
| 49 | sfaD, sfaX, sfaY | - | - | - | - | - | - | - | - | - | - | - | + | - | - | - | 1 |
| 50 | sfaF | - | - | - | - | + | - | - | - | - | - | - | - | - | - | - | 1 |
| 51 | tcpC | - | - | - | - | + | - | - | - | - | - | - | + | - | - | - | 2 |
| 52 | Vat | - | - | - | - | + | + | - | - | - | - | - | + | - | - | - | 3 |
| 53 | yagV/ecpE,yagW/ecpD,yagX/ecpC,yagY/ecpB,yagZ/ecpA | + | - | + | + | + | + | + | + | + | + | + | + | + | + | + | 14 |
| 54 | ybtA, ybtE, ybtP ,ybtQ, ybtS, ybtT, ybtU, ybtX | + | + | + | - | + | + | + | + | + | + | + | + | + | + | - | 13 |
| 55 | ykgK/ecpR | + | - | + | + | + | + | + | + | + | + | + | + | + | + | + | 14 |
| Total | | 23 | 17 | 18 | 14 | 37 | 29 | 29 | 24 | 23 | 29 | 31 | 37 | 21 | 24 | 16 | |

*, Abricate virulence finder database (https://github.com/tseemann/abricate) was used for the presence (+) or absence (-) of a gene.

**Table S5.** Comparison of virulent genes in the 10 ocular *E. coli* isolates and *E. coli* type strains associated with phylogenomic groups A to F

| Sl. No. | Gene | A | | B1 | B2 | | | | | | | C | D | E | F |
|---|---|---|---|---|---|---|---|---|---|---|---|---|---|---|---|
| | | L-2594/2017 | L-3003/2015 | ETEC 2886-350 | L-1339/2013 | L-494/2011 | L-3137/2017 | L-1149/2016 | GMRV-476/2017 | L-1534/2016 | L-1216/2010 | L-1010/2018 | ec042 | SAK-AI | IAI-39 |
| 1 | *afaA, afaB-I, afaC-I, afaD, afaE-I* | - | - | - | - | - | - | - | - | - | - | + | - | - | - |
| 2 | *aslA* | + | + | - | + | + | + | + | + | + | + | - | + | + | + |
| 3 | *astA* | - | - | - | - | - | - | - | - | - | - | - | - | + | - |
| 4 | *cesAB, cesD, cesD2, cesF, cesL, cesT* | - | - | - | - | - | - | - | - | - | - | - | - | + | - |
| 6 | *chuA* | - | - | - | + | + | + | + | + | + | + | - | - | - | - |
| 7 | *chuS, chuT* | - | - | - | + | + | + | + | + | + | + | - | - | - | + |
| 8 | *chuU, chuV* | - | - | - | + | + | + | + | + | + | + | - | + | + | + |
| 9 | *chuW* | - | - | - | + | + | + | + | + | + | + | - | + | + | + |
| 10 | *chuX* | - | - | - | + | + | + | + | + | + | + | - | - | - | - |
| 11 | *chuY* | - | - | - | + | + | + | + | + | + | + | - | + | - | + |
| 12 | *csfA, cssA* | - | - | + | - | - | - | - | - | - | - | - | - | - | - |
| 13 | *daaF, draP* | - | - | - | - | - | - | - | - | - | - | + | - | - | - |
| 14 | *eltA, eltB* | - | - | + | - | - | - | - | - | - | - | - | - | - | - |
| 15 | *entA, entB, entC* | + | + | + | + | + | + | + | + | + | + | + | + | + | + |
| 16 | *entD* | + | + | + | - | - | - | - | - | - | + | + | + | + | + |
| 17 | *entE, entF, entS* | + | + | + | + | + | + | + | + | + | + | + | + | + | + |
| 18 | *escC, escD, escE, escF, escG, escI, escJ, escL, escN, escO, escP, escR, escS, escT, escU, escV, espA, espB, espD, espF, espG, espH, espJ, espK* | - | - | - | - | - | - | - | - | - | - | - | - | + | - |
| 19 | *espL1* | + | + | + | - | - | - | - | - | - | - | + | + | + | + |
| 20 | *espL2* | - | - | - | - | - | - | - | - | - | - | - | - | + | - |

| **Sl. No.** | **Gene** | **A** | | **B1** | **B2** | | | | | | | C | D | E | F |
|---|---|---|---|---|---|---|---|---|---|---|---|---|---|---|---|
| | | L-2594/2017 | L-3003/2015 | ETEC 2886-350 | L-1339/2013 | L-494/2011 | L-3137/2017 | L-1149/2016 | GMRV-476/2017 | L-1534/2016 | L-1216/2010 | L-1010/2018 | ec042 | SAK-AI | IAI-39 |
| 21 | *espL4* | + | + | - | - | - | - | - | - | - | - | - | + | + | - |
| 22 | *espM1, espM2* | - | - | - | - | - | - | - | - | - | - | - | - | + | - |
| 23 | *espN, espP* | - | - | - | - | - | - | - | - | - | - | - | - | + | - |
| 24 | *espR1* | - | - | - | - | - | - | - | - | - | - | - | + | + | + |
| 25 | *espR3, espR4* | - | - | - | - | - | - | - | - | - | - | - | - | + | - |
| 26 | *espW* | - | - | - | - | - | - | - | - | - | - | - | - | + | - |
| 27 | *espX2* | - | - | - | - | - | - | - | - | - | - | - | - | + | + |
| 28 | *espX4* | + | + | + | - | - | - | - | - | - | - | + | + | + | + |
| 29 | *espX5* | + | + | + | - | - | - | - | - | - | - | + | + | + | - |
| 30 | *espX6, espX7/nleL* | - | - | - | - | - | - | - | - | - | - | - | - | + | - |
| 31 | *espY1* | - | - | - | - | - | - | - | - | - | - | - | + | + | - |
| 32 | *espY2* | - | - | - | - | - | - | - | - | - | - | - | + | + | + |
| 33 | *espY3, espY4* | - | - | - | - | - | - | - | - | - | - | - | + | + | - |
| 34 | *etgA* | - | - | - | - | - | - | - | - | - | - | - | - | + | - |
| 35 | *fdeC, fepA, fepB, fepC, fepD, fepG, fes* | + | + | + | + | + | + | + | + | + | + | + | + | + | + |
| 36 | *fimA* | - | - | + | + | + | + | + | + | + | + | + | - | + | + |
| 37 | *fimB* | - | + | + | - | + | + | - | - | + | + | + | - | + | + |
| 38 | *fimC, fimD, fimE, fimF, fimG, fimH, fimI* | - | + | + | + | + | + | + | + | + | + | + | - | + | + |
| 39 | *focA, focF, focG, focH* | - | - | - | - | - | - | - | - | - | + | - | - | - | - |
| 40 | *fyuA* | + | + | - | + | + | + | + | + | + | + | + | + | - | + |
| 41 | *gspC, gspD, gspE, gspF, gspG, gspH, gspI, gspJ, gspK, gspL, gspM* | - | - | + | - | + | + | + | - | + | + | - | + | - | + |
| 42 | *hlyA, hlyB, hlyC, hlyD* | - | - | - | - | + | + | + | - | - | + | - | - | + | - |

| Sl. No. | Gene | A | | B1 | B2 | | | | | | | C | D | E | F |
|---|---|---|---|---|---|---|---|---|---|---|---|---|---|---|---|
| | | L-2594/2017 | L-3003/2015 | ETEC 2886-350 | L-1339/2013 | L-494/2011 | L-3137/2017 | L-1149/2016 | GMRV-476/2017 | L-1534/2016 | L-1216/2010 | L-1010/2018 | ec042 | SAK-AI | IAI-39 |
| 43 | *iroB, iroC, iroD, iroE, iroN* | - | - | - | - | - | - | - | - | - | + | - | - | - | - |
| 44 | *irp1, irp2* | + | + | - | + | + | + | + | + | + | + | + | + | - | + |
| 45 | *iucA, iucB* | + | + | - | + | + | + | + | + | + | - | + | - | - | + |
| 46 | *iucC* | + | + | - | + | - | + | + | - | + | - | + | - | - | + |
| 47 | *iucD* | + | + | - | + | + | + | + | + | + | - | + | - | - | + |
| 48 | *iutA* | - | - | - | - | - | - | - | + | - | - | - | - | - | - |
| 49 | *kpsD, kpsM* | - | - | - | + | + | + | + | + | + | + | - | + | - | + |
| 50 | *kpsT* | - | - | - | - | - | - | - | - | + | - | - | - | - | + |
| 51 | *map, nleA/espI, nleB1, nleB2, nleC, nleD, nleE, nleF, nleG7, nleH1, nleH2* | - | - | - | - | - | - | - | - | - | - | - | - | + | - |
| 52 | *ompA* | + | + | + | + | + | + | + | + | + | + | + | + | + | + |
| 53 | *paa* | - | - | - | - | - | - | - | - | - | - | - | - | + | - |
| 54 | *papB, papC, papD,* | + | - | - | - | + | + | - | + | + | + | - | - | - | + |
| 55 | *papE* | - | - | - | - | - | - | - | - | - | - | - | - | - | + |
| 56 | *papF, papG, papH* | + | - | - | + | + | + | + | + | - | + | - | - | - | + |
| 57 | *papI* | + | - | - | + | + | + | + | + | + | + | - | - | - | + |
| 58 | *papJ* | + | - | - | + | + | + | + | + | - | + | - | - | - | + |
| 59 | *papK* | + | - | - | + | + | + | + | - | - | + | - | - | - | + |
| 60 | *papX* | + | - | - | + | + | + | + | + | + | - | - | - | - | + |
| 61 | *pic* | - | - | - | - | - | - | - | - | - | + | - | + | - | - |
| 62 | *sat* | - | - | - | + | + | + | + | + | + | - | - | - | - | - |
| 63 | *senB* | - | - | - | - | - | + | - | - | + | + | + | - | - | - |
| 64 | *sepD, sepL, sepQ/escQ, sepZ/escZ* | - | - | - | - | - | - | - | - | - | - | - | - | + | - |

| Sl. No. | Gene | A | | B1 | B2 | | | | | | | C | D | E | F |
|---|---|---|---|---|---|---|---|---|---|---|---|---|---|---|---|
| | | L-2594/2017 | L-3003/2015 | ETEC 2886-350 | L-1339/2013 | L-494/2011 | L-3137/2017 | L-1149/2016 | GMRV-476/2017 | L-1534/2016 | L-1216/2010 | L-1010/2018 | ec042 | SAK-AI | IAI-39 |
| 65 | *sfaB, sfaC, sfaF* | - | - | - | - | - | - | - | - | - | + | - | - | - | - |
| 66 | *shuA* | - | - | - | - | - | - | - | - | - | - | - | + | + | + |
| 67 | *shuS, shuT* | - | - | - | - | - | - | - | - | - | - | - | + | + | - |
| 68 | *shuX* | - | - | - | - | - | - | - | - | - | - | - | + | + | + |
| 69 | *shuY, stcE, stx1A, stx1B, stx2A, stx2B* | - | - | - | - | - | - | - | - | - | - | - | - | + | - |
| 70 | *tcpC* | - | - | - | - | - | - | - | - | - | + | - | - | - | - |
| 71 | *tir, toxB* | - | - | - | - | - | - | - | - | - | - | - | - | + | - |
| 72 | *vat* | - | - | - | - | - | - | - | - | + | + | - | - | - | - |
| 73 | *yagV/ecpE, yagW/ecpD, yagX/ecpC, yagY/ecpB, yagZ/ecpA* | + | - | + | + | + | + | + | + | + | + | + | + | + | + |
| 74 | *ybtA, ybtE, ybtP, ybtQ, ybtS, ybtT, ybtU, ybtX* | + | + | - | + | + | + | + | + | + | + | + | + | - | + |
| 75 | *ykgK/ecpR* | + | - | + | + | + | + | + | + | + | + | + | + | + | + |

*, Abricate virulence finder database (https://github.com/tseemann/abricate) was used for the presence (+) or absence (-) of a gene.

**Table S6.** Number of prophages* in ocular *E. coli* isolates.

| Sl. No. | Prophage | L-1339/2013 | L-2594/2017 | L-494/2011 | L-3003/2015 | L-1010/2018 | L-3137/2017 | L-1149/2016 | GMR V-476/2017 | L-1534/2016 | L-1216/2010 | K-12 MG1655 | Count |
|---|---|---|---|---|---|---|---|---|---|---|---|---|---|
| **1** | *Bacillus* phage G | 0 | 0 | 0 | 1 | 0 | 0 | 0 | 0 | 0 | 0 | 0 | 1 |
| **2** | *Burkholderia* phage phiE255 | 0 | 0 | 0 | 0 | 0 | 0 | 0 | 1 | 0 | 0 | 0 | 1 |
| **3** | Enterobacteria phage 186 | 0 | 1 | 0 | 0 | 0 | 0 | 0 | 0 | 0 | 0 | 0 | 1 |
| **4** | Enterobacteria phage BP-4795 | 1 | 0 | 2 | 0 | 0 | 1 | 1 | 0 | 1 | 0 | 0 | 6 |
| **5** | Enterobacteria phage fiAA91-ss | 0 | 0 | 0 | 0 | 0 | 0 | 0 | 1 | 0 | 0 | 0 | 1 |
| **6** | Enterobacteria phage HK544 | 0 | 1 | 0 | 0 | 0 | 0 | 0 | 0 | 0 | 1 | 0 | 2 |
| **7** | Enterobacteria phage HK97 | 0 | 0 | 1 | 0 | 0 | 0 | 0 | 0 | 0 | 0 | 0 | 1 |
| **8** | Enterobacteria phage lambda | 1 | 0 | 1 | 1 | 0 | 1 | 2 | 1 | 1 | 1 | 2 | 11 |
| **9** | Enterobacteria phage mEp460 | 1 | 1 | 1 | 3 | 1 | 1 | 0 | 1 | 0 | 1 | 0 | 10 |
| **10** | Enterobacteria phage P1 | 1 | 1 | 0 | 0 | 0 | 0 | 0 | 0 | 0 | 0 | 0 | 2 |
| **11** | Enterobacteria phage P2 | 0 | 0 | 1 | 0 | 0 | 0 | 0 | 0 | 1 | 0 | 0 | 2 |
| **12** | Enterobacteria phage P4 | 1 | 0 | 0 | 0 | 0 | 1 | 0 | 0 | 1 | 0 | 0 | 3 |
| **13** | Enterobacteria phage P88 | 0 | 0 | 2 | 0 | 0 | 2 | 1 | 1 | 0 | 1 | 0 | 7 |
| **14** | *Escherichia* Stx1 converting phage | 0 | 0 | 0 | 0 | 0 | 0 | 0 | 0 | 1 | 0 | 0 | 1 |
| **15** | *Escherichia* phage D108 | 0 | 0 | 1 | 0 | 0 | 0 | 0 | 0 | 0 | 0 | 0 | 1 |
| **16** | *Escherichia* phage TL-2011b | 0 | 0 | 0 | 0 | 0 | 0 | 0 | 1 | 1 | 0 | 0 | 2 |
| **17** | *Escherichia* phage vB_EcoM-ep3 | 0 | 0 | 0 | 0 | 0 | 1 | 0 | 0 | 0 | 0 | 0 | 1 |
| **18** | *Pectobacterium* phage ZF40 | 1 | 0 | 1 | 0 | 0 | 0 | 1 | 1 | 0 | 0 | 0 | 4 |
| **19** | *Salmonella* phage 118970_sal3 | 0 | 0 | 0 | 0 | 0 | 0 | 0 | 0 | 1 | 0 | 1 | 2 |
| **20** | *Salmonella* phage SEN34 | 0 | 0 | 0 | 0 | 0 | 0 | 0 | 0 | 0 | 1 | 0 | 1 |
| **21** | *Salmonella* phage SJ46 | 0 | 0 | 1 | 0 | 0 | 0 | 0 | 1 | 0 | 0 | 0 | 2 |
| **22** | *Shigella* phage SfIV | 0 | 0 | 0 | 0 | 0 | 0 | 0 | 0 | 0 | 0 | 1 | 1 |
| **23** | Stx2-converting phage 1717 | 1 | 1 | 0 | 1 | 0 | 0 | 0 | 0 | 0 | 0 | 0 | 3 |
| **24** | *Yersinia* phage L-413C | 1 | 0 | 0 | 0 | 0 | 0 | 0 | 0 | 0 | 0 | 0 | 1 |
| | **Total** | 8 | 5 | 9 | 4 | 1 | 6 | 4 | 8 | 7 | 5 | 3 | |

*, Prophages were detected using PHASTER 4.3 (http://phaster.ca/).

**Table S7.** Comparison of prophages in ocular, EPEC, ETEC, ExPEC, UPEC and environmental *E. coli* strains

| Sl. No. | Prophage | OCULAR | EPEC | ETEC | ExPEC | UPEC | Environmental |
|---|---|---|---|---|---|---|---|
| | | n=10 | n=23 | n=25 | n=14 | n=5 | n=21 |
| 1 | *Aeromonas* phage phiO18P | 0 | 0 | 1 | 0 | 0 | 1 |
| 2 | *Bacillus* phage G | 1 | 0 | 0 | 0 | 0 | 0 |
| 3 | Bacteriophage APSE-2 | 0 | 0 | 1 | 0 | 0 | 0 |
| 4 | *Burkholderia* phage BcepMu | 0 | 1 | 0 | 0 | 0 | 0 |
| 5 | *Burkholderia* phage phiE255 | 1 | 0 | 1 | 4 | 0 | 0 |
| 6 | *Edwardsiella* phage GF-2 | 0 | 1 | 0 | 0 | 0 | 0 |
| 7 | Enterobacteria phage 186 | 1 | 0 | 0 | 0 | 1 | 0 |
| 8 | Enterobacteria phage 933W | 0 | 0 | 0 | 1 | 0 | 0 |
| 9 | Enterobacteria phage BP-4795 | 5 | 13 | 10 | 10 | 0 | 3 |
| 10 | Enterobacteria phage cdtI | 0 | 6 | 4 | 0 | 0 | 2 |
| 11 | Enterobacteria phage fiAA91-ss | 1 | 3 | 4 | 2 | 0 | 1 |
| 12 | Enterobacteria phage HK022 | 0 | 0 | 0 | 2 | 0 | 0 |
| 13 | Enterobacteria phage HK544 | 2 | 0 | 0 | 2 | 0 | 0 |
| 14 | Enterobacteria phage HK629 | 0 | 3 | 3 | 0 | 0 | 1 |
| 15 | Enterobacteria phage HK630 | 0 | 3 | 0 | 0 | 1 | 2 |
| 16 | Enterobacteria phage HK97 | 1 | 0 | 0 | 0 | 0 | 0 |
| 17 | Enterobacteria phage I2-2 | 0 | 0 | 1 | 0 | 0 | 0 |
| 18 | Enterobacteria phage IME10 | 0 | 0 | 2 | 0 | 0 | 0 |
| 19 | Enterobacteria phage lambda | 8 | 21 | 18 | 11 | 5 | 8 |
| 20 | Enterobacteria phage phiX174 sensu lato | 0 | 0 | 1 | 0 | 0 | 0 |
| 21 | Enterobacterial phage mEp390 | 0 | 0 | 1 | 0 | 0 | 0 |
| 22 | Enterobacteria phage mEp460 | 8 | 9 | 7 | 4 | 3 | 2 |
| 23 | Enterobacteria phage Mu | 0 | 0 | 0 | 1 | 0 | 4 |
| 24 | Enterobacteria phage N15 | 0 | 0 | 0 | 0 | 0 | 1 |
| 25 | Enterobacteria phage P1 | 2 | 0 | 0 | 0 | 0 | 0 |
| 26 | Enterobacteria phage P2 | 2 | 3 | 1 | 1 | 1 | 1 |
| 29 | Enterobacteria phage phiP27 | 0 | 1 | 2 | 1 | 0 | 0 |
| 30 | Enterobacteria phage PsP3 | 0 | 1 | 5 | 0 | 0 | 0 |
| 31 | Enterobacteria phage Sf101 | 0 | 2 | 2 | 0 | 0 | 1 |
| 32 | Enterobacteria phage SfI | 0 | 4 | 1 | 0 | 1 | 0 |
| 33 | Enterobacteria phage SfV | 0 | 4 | 2 | 0 | 0 | 0 |
| 34 | Enterobacteria phage WPhi | 0 | 1 | 2 | 0 | 0 | 1 |
| 35 | Enterobacteria phage YYZ-2008 | 0 | 4 | 0 | 0 | 0 | 1 |
| 36 | *Escherichia* phage D108 | 1 | 1 | 1 | 0 | 0 | 1 |
| 37 | *Escherichia* phage HK639 | 0 | 0 | 1 | 4 | 0 | 0 |
| 38 | *Escherichia* Stx1 converting phage | 1 | 0 | 0 | 0 | 0 | 0 |
| 39 | *Escherichia* phage phiV10 | 0 | 0 | 2 | 1 | 0 | 0 |
| 40 | *Escherichia* phage TL-2011b | 2 | 0 | 1 | 3 | 1 | 0 |
| 41 | *Escherichia* phage pro147 | 0 | 0 | 0 | 1 | 0 | 0 |
| 42 | *Escherichia* phage pro483 | 0 | 2 | 2 | 1 | 1 | 0 |
| 43 | *Escherichia* phage vB_EcoM_ECO1230-10 | 0 | 0 | 1 | 0 | 0 | 1 |
| 44 | *Escherichia* phage vB_EcoM-ep3 | 1 | 0 | 0 | 0 | 0 | 0 |
| 45 | Phage Gifsy-1 | 0 | 3 | 1 | 0 | 1 | 0 |
| 46 | Haemophilus phage SuMu | 0 | 1 | 0 | 0 | 0 | 0 |
| 47 | *Pectobacterium* phage ZF40 | 4 | 0 | 0 | 5 | 0 | 0 |
| 48 | *Pseudomonas* phage phiPSA1 | 0 | 0 | 0 | 0 | 0 | 1 |
| 49 | *Pseudomonas* phage PPpW-3 | 0 | 0 | 0 | 0 | 0 | 2 |
| 50 | *Salmonella* phage 118970_sal3 | 1 | 3 | 2 | 0 | 0 | 3 |
| 53 | *Salmonella* phage SEN34 | 1 | 1 | 4 | 1 | 0 | 0 |
| 54 | *Salmonella* phage SJ46 | 2 | 2 | 1 | 1 | 0 | 0 |
| 55 | *Salmonella* phage FSL SP-004 | 0 | 0 | 0 | 1 | 0 | 0 |
| 56 | *Salmonella* phage SPN1S | 0 | 1 | 0 | 0 | 0 | 0 |
| 57 | *Salmonella* phage SSU5 | 0 | 5 | 1 | 0 | 0 | 1 |
| 58 | *Salmonella* phage vB_SosS_Oslo | 0 | 0 | 1 | 0 | 0 | 0 |
| 61 | *Shigella* phage SfIV | 0 | 1 | 3 | 0 | 1 | 1 |
| 62 | *Staphylococcus* phage SPbeta-like | 0 | 0 | 1 | 0 | 0 | 0 |
| 63 | Stx2-converting phage 1717 | 3 | 3 | 11 | 5 | 0 | 1 |
| 64 | *Vibrio* phage martha 12B12 | 0 | 0 | 0 | 0 | 0 | 1 |
| 65 | *Vibrio* phage X29 | 0 | 0 | 1 | 0 | 0 | 0 |
| 66 | *Yersinia* phage L-413C | 1 | 0 | 0 | 1 | 0 | 0 |

*, Prophages were detected using PHASTER 4.3 (http://phaster.ca/)

**Table S8.** Number of quorum sensing genes * in ocular *E. coli* isolates.

| Sl. No. | Gene | L-1339/2013 | L-2594/2017 | L-494/2011 | L-3003/2015 | L-1010/2018 | L-1037/2017 | L-1149/2016 | GMRV-457/2017 | L-1534/2016 | L-1216/2010 |
|---|---|---|---|---|---|---|---|---|---|---|---|
| **1** | *lsrK* | + | + | + | + | - | - | - | - | - | - |
| **2** | *luxS* | + | - | + | - | - | - | - | - | + | + |
| **3** | *lsrB* | - | + | - | + | + | - | - | - | - | - |
| **4** | *lsrC* | - | + | - | + | - | - | - | - | - | - |
| **5** | *lsrR* | - | + | - | - | - | - | - | - | - | - |
| **6** | *lsrD* | - | + | - | - | - | - | - | - | - | - |
| **7** | *lsrG* | - | + | - | - | - | - | - | - | - | - |
| **Total** | | 2 | 6 | 2 | 3 | 1 | 0 | 0 | 0 | 1 | 1 |

*, Quorum sensing genes were detected using (+) and (-) indicate presence and absence of genes respectively.

**Table S9.** Biofilm genes*in ocular *E. coli* isolates.

| Sl. No. | Gene | L-1339/2013 | L-2594/2017 | L-494/2011 | L-3003/2015 | L-1010/2018 | L-1037/2017 | L-1149/2016 | GMRV-457/2017 | L-1534/2016 | L-1216/2010 | Count |
|---|---|---|---|---|---|---|---|---|---|---|---|---|
| **1** | *bdcA* | - | + | - | + | - | - | - | - | - | - | 2 |
| **2** | *csgD* | + | + | + | + | - | - | - | - | - | + | 5 |
| **3** | *csrA* | + | + | + | + | - | - | - | - | + | + | 6 |
| **4** | *csrD* | + | - | + | - | - | - | - | - | - | - | 2 |
| **5** | *dnaK* | - | + | - | - | - | - | - | - | - | - | 1 |
| **6** | *flhC* | + | + | + | + | + | + | + | + | - | + | 9 |
| **7** | *mqsR* | - | + | - | - | - | - | - | - | - | - | 1 |
| **8** | *nhaR* | - | + | - | - | - | - | - | - | - | + | 2 |
| **9** | *ompF* | + | + | + | + | - | - | - | - | - | + | 5 |
| **10** | *rcsA* | + | - | + | - | - | - | - | - | - | - | 2 |
| **11** | *rcsB* | + | + | + | + | - | - | - | - | - | + | 5 |
| **12** | *rcsD* | + | - | + | - | - | - | - | - | - | + | 3 |
| **13** | *relA* | + | + | + | + | - | - | - | - | + | + | 6 |
| **14** | *rpoS* | + | - | + | + | - | - | - | - | - | + | 4 |
| **15** | *spoT* | + | + | + | + | - | - | - | - | + | + | 6 |
| **16** | *tolA* | - | + | - | - | + | - | - | - | + | + | 4 |
| **Total** | | 11 | 12 | 11 | 9 | 2 | 1 | 1 | 1 | 4 | 11 | |

*, Biofilm genes were detected using (+) and (–) indicate presence and absence of genes respectively.

**Table S10.** Motility genes* in ocular *E. coli* isolates.

| **Sl. No.** | **Gene** | **L-339/2013** | **L-2594/2017** | **L-494/2011** | **L-3003/2015** | **L-1010/2018** | **L-3137/2017** | **L-1149/2016** | **GMRV-476/2017** | **L-1534/2016** | **L-1216/2010** | **Count** |
|---|---|---|---|---|---|---|---|---|---|---|---|---|
| **1** | *atoC* | + | + | + | - | - | - | - | - | - | + | 4 |
| **2** | *atoS* | + | - | + | - | - | - | - | - | + | - | 3 |
| **3** | *barA* | + | + | + | + | - | - | - | - | + | + | 6 |
| **4** | *cheA* | + | + | + | + | - | - | - | - | + | + | 6 |
| **5** | *cheY* | + | + | + | + | - | - | - | - | + | + | 6 |
| **6** | *cpxA* | + | + | + | + | - | - | - | - | + | + | 6 |
| **7** | *cpxR* | + | - | + | - | - | - | - | - | + | + | 4 |
| **8** | *crp* | - | + | - | - | - | - | - | - | - | - | 1 |
| **9** | *csrA* | + | + | + | + | - | - | - | - | + | + | 6 |
| **10** | *dnaJ* | + | + | + | + | + | - | - | - | + | + | 7 |
| **11** | *dnaK* | + | - | + | + | - | - | - | - | + | + | 5 |
| **12** | *envZ* | + | + | + | + | + | + | + | + | + | + | 10 |
| **13** | *flgK* | + | - | + | - | - | - | - | - | - | - | 2 |
| **14** | *flgM* | + | - | + | - | - | - | - | - | + | + | 4 |
| **15** | *fliA* | + | - | + | - | - | - | - | - | + | - | 3 |
| **16** | *fliD* | + | - | + | - | - | - | - | - | - | - | 2 |
| **17** | *hdfR* | + | + | + | + | - | + | + | + | + | + | 9 |
| **18** | *lrhA* | + | + | + | + | - | - | - | - | + | + | 6 |
| **19** | *matA* | + | - | + | + | - | - | - | - | + | + | 5 |
| **20** | *motA* | + | + | + | + | - | - | - | - | + | + | 6 |
| **21** | *mqsR* | - | + | - | - | - | - | - | - | + | - | 2 |
| **22** | *nsrR* | - | - | - | - | - | - | - | - | + | - | 1 |
| **23** | *ompR* | + | + | + | + | - | - | - | - | + | + | 6 |
| **24** | *rcsA* | + | - | + | - | - | - | - | - | - | - | 2 |
| **25** | *rcsC* | - | + | + | + | - | - | - | - | - | + | 4 |
| **26** | *rcsD* | + | - | + | - | - | - | - | - | + | + | 4 |
| **27** | *tsr* | + | + | - | + | - | - | - | - | - | - | 3 |
| **28** | *uvrY* | + | - | + | - | - | - | - | - | + | + | 4 |
| **29** | *ydiV* | + | - | + | - | - | - | - | - | - | + | 3 |
| **Total** | | 25 | 16 | 25 | 15 | 2 | 2 | 2 | 2 | 21 | 20 | |

*, Motility genes were detected using (+) and (–) indicate presence and absence of genes respectively.

Table S11. Characteristics of patients from whom the 10 ocular *E. coli* were isolated

| **Serial no.** | **Lab. no.** | **Age/ gender** | **Eye** | **Type of sample** | **Clinical Diagnosis** | **Symptoms** | **Visual acuity at** | |
|---|---|---|---|---|---|---|---|---|
| | | | | | | | **Present-ation** | **Last visit** |
| 1 | L-1339/2013 | 1/M | OS | Conjunctival swab | Conjunctivitis | Redness, watering | FAFL | FAFL |
| 2 | L-1216/2010 | 58/M | OS | Vitreous | Endophthalmitis | Pain, diminution of vision | LP | 20/25 |
| 3 | L-494/2011 | 40/M | OS | Vitreous | Endophthalmitis | Pain, diminution of vision | 20/50 | 20/30 |
| 4 | L-2594/2017 | 61/F | OS | Vitreous | Endophthalmitis | Sudden painless vision loss in the left eye | 20/30 | No LP |
| 5 | L-3003/2015 | 4/F | OD | Corneal scraping | Infective keratitis | diminution of vision | FFL | Not recorded |
| 6 | L-1534/2017 | 77/M | OS | Conjunctival swab | Conjunctivitis | Pain, Redness and swollen eye | No LP | No LP |
| 7 | L-1010/2018 | 25/F | OD | Pus | Orbital Cellulitis | Protrusion of right eye ball, redness and discharge | No LP | Not recorded |
| 8 | L-1149/2016 | 47/F | OD | Corneal scraping | Infective keratitis | Pain, discharge | LP inaccurate | HM+ |
| 9 | L-3137/2017 | 65/F | OS | Vitreous | Endophthalmitis | Pain, diminution of vision | LP | LP inaccurate |
| 10 | GMRV-476/2017 | 56/M | OD | Vitreous | Endophthalmitis | Pain, Redness and swollen eye | LP inaccurate | No LP |

HM- Hand movement, FAFL-Fixing and following light, LP- light Perception , FFL- Fixing and following light, PR-Perception of Rays, OS: Oculus sinister; OD: Oculus dexter; M: Male; F: Female;
